# Effect of plasma exchange in neuromyelitis optica spectrum disorder: A systematic review and meta‐analysis

**DOI:** 10.1002/acn3.51203

**Published:** 2020-09-21

**Authors:** Punchika Kosiyakul, Sakdipat Songwisit, Patompong Ungprasert, Sasitorn Siritho, Naraporn Prayoonwiwat, Jiraporn Jitprapaikulsan

**Affiliations:** ^1^ Faculty of Medicine Siriraj Hospital Mahidol University Bangkok Thailand; ^2^ Clinical Epidemiology Unit Department of Development Faculty of Medicine Siriraj Hospital Mahidol University Bangkok Thailand; ^3^ Department of Rheumatic & Immunologic Diseases Cleveland Clinic Cleveland Ohio USA; ^4^ Siriraj Neuroimmunology Center Faculty of Medicine Siriraj Hospital Mahidol University Bangkok Thailand; ^5^ Bumrungrad Hospital Bangkok Thailand; ^6^ Division of Neurology Department of Medicine Faculty of Medicine Siriraj Hospital Mahidol University Bangkok Thailand

## Abstract

**Objective:**

To conduct systematic review and meta‐analysis for the efficacy of therapeutic plasma exchange (TPE) for neuromyelitis optica spectrum disorder (NMOSD) with an acute attack.

**Methods:**

Systematic review was performed using EMBASE and OVID/Medline database. The eligible studies must be the studies of NMOSD patients treated with TPE during the acute phase. They must report treatment outcomes using either Expanded Disability Status Scale (EDSS) or visual acuity (VA) before and after the therapy. Pooled mean difference (MD) was then calculated by combining MDs of each study using the random‐effects model.

**Results:**

Fifteen studies were identified; eleven with 241 NMOSD patients reported EDSS outcome and four studies with 103 NMOSD reported visual outcomes. The meta‐analysis demonstrated a significantly decreased in EDSS after TPE treatment for NMOSD with an acute attack with the pooled MD of 0.83 (95% CI, 0.26–1.40; I^2^ 69%) comparing pretreatment to immediate posttreatment and 2.13 (95% CI, 1.55–2.70; I^2^ 31%) comparing pretreatment to posttreatment at 6 months to 1‐year follow‐up. Unfortunately, only one of the four studies evaluating visual outcomes reported standard deviation in association with mean LogMAR; therefore, the meta‐analysis cannot be conducted. Nonetheless, all studies consistently demonstrated the benefit of TPE with improved VA and/or LogMAR after treatment.

**Interpretation:**

This systematic review and meta‐analysis showed the benefit of TPE during the NMOSD attack with a significantly improved disability status immediately after treatment and during follow‐up.

## Introduction

Neuromyelitis optica spectrum disorder (NMOSD) is an autoimmune demyelinating disorder of the central nervous system (CNS) caused by the binding of aquaporin‐4 (AQP4)‐immunoglobulin (IgG) to water channel protein at astrocyte foot process. Common manifestations of NMOSD include transverse myelitis, optic neuritis, area postrema syndrome, and brainstem syndrome.^1^ There is no specific measurement outcome but generally measured using the Expanded Disability Status Scale (EDSS) and visual acuity (VA) for disability. The severity of the first attack and the number of relapses in the first 2 years are the most important prognostic factors.^1^ Therefore, early aggressive treatment after the initial attacks is generally considered an important measure that could heavily influence the long‐term prognosis of patients with NMOSD. With the lack of data from randomized controlled trials, the current treatment strategy for an acute attack of NMOSD is derived from clinical studies and experiences of other acute demyelinating diseases. High‐dose corticosteroids are generally considered as the standard first‐line therapy, although the role of therapeutic plasma exchange (TPE) or plasmapheresis has increasingly been recognized.^2^, ^3^ Plasma exchange eliminates circulating antibodies and inflammatory cytokines, which are the principal mediators of NMOSD attack.^4^ Several nonrandomized studies have suggested the role of TPE as rescue therapy or even first‐line therapy.^5^, ^6^, ^7^, ^8^, ^9^, ^10^, ^11^, ^12^ Nonetheless, the number of those studies is still relatively limited, and the results are somewhat inconsistent. This systematic review and meta‐analysis were conducted to evaluate the efficacy of TPE in NMOSD patients with an acute attack.

## Methods

### Literature search strategy

Two investigators (P.K. and S.S.) independently searched for eligible articles in EMBASE and OVID/Medline database from inception to October 2019 using the search strategy included the terms related to NMOSD combined with the terms related to plasma exchange. The detailed search strategy is provided in Data S1. References of retrieved articles were also manually reviewed for additional eligible studies. This study was undertaken in accordance with the Preferred Reporting Items for Systematic Reviews and Meta‐Analyses (PRISMA) statement, which is available as File S1.

### Inclusion and exclusion criteria for the literature

The eligible studies must be the studies of NMOSD patients treated with TPE during the acute phase including all clinical attacks. The study must report treatment outcomes using either EDSS or VA before and after the therapy. Case report and case series of fewer than three patients were not included to minimize the chance of selecting nonrepresentative cases into the meta‐analysis.

All retrieved articles were independently reviewed by the first two investigators (P.K. and S.S.) for their eligibility. The eligible studies were also reviewed by two senior investigators (P.U. and J.J.) to ensure that inclusion criteria were met. The senior investigators also helped to decide the eligibility of the study when the first two investigators made different determinations. The data discrepancy was reviewed, discussed, and made a consensus.

### Data extraction

A standardized data collection form was used to extract the following information; the name of the first author, year of publication, country of origin, type of study design, method (s) used to identify/recruit patients into the study, age at onset/diagnosis, characteristics of NMOSD attacks, the total number of patients and number of patients with positive AQP4 antibody and positive MOG antibody, number of the patient receiving TPE, number of attacks and total affected eyes or number of transverse myelitis attacks, demographic data of the patients, TPE regimen, use of other immunosuppressive therapy, duration of follow‐up and treatment, pre and posttreatment EDSS and pre and posttreatment VA. This data extraction was also independently conducted by the first two investigators. Data discrepancy was reviewed and discussed. The final consensus was made by referring to the original articles.

### Statistical analysis

Statistical analysis was performed using Review Manager 5.3 software from the Cochrane Collaboration (London, United Kingdom). Mean pre and posttreatment EDSS, as well as mean pre and posttreatment Logarithm of the Minimum Angle of Resolution (LogMAR), along with their standard deviation (SD), were extracted from each study and the mean difference (MD) was calculated. Pooled MD was then calculated by combining MDs of each study using the random‐effects model. If the study described the median and interquartile range (IQR) instead of mean and SD, the median would be used as an estimate for mean, and SD would be estimated from IQR/1.35.

Statistical heterogeneity was evaluated using Cochran's Q test, which is complemented with the I^2^ statistic, which quantifies the proportion of the total variation across studies that is due to heterogeneity rather than chance. A value of I^2^ of 0–25% represents insignificant heterogeneity, 26–50% low heterogeneity, 51–75% moderate heterogeneity, and >75% high heterogeneity.^13^

Publication bias would be assessed by the visualization of the funnel plot if enough studies were eligible for the meta‐analysis.

## Results

### Study identification and selection

The systematic search strategy identified 1336 potentially relevant articles (1082 from EMBASE and 254 articles from OVID/MEDLINE). After the exclusion of 199 duplicated articles, 1137 articles underwent title and abstract review.

A total of 1063 articles were excluded at this stage as they did not fulfill the eligibility criteria based on the type of article, study design, and outcome of interest. The remaining 74 articles underwent a full‐length article review. After the full‐length review, 59 articles were excluded as the outcomes of interest were not reported, resulting in 15 eligible studies. Across those 15 studies, 11 studies^5^, ^6^, ^7^, ^21^ with 241 NMOSD patients (>146 [60.9%] were AQP4 IgG seropositive patients) reported EDSS scores before and after the TPE, while the other four studies^9^, ^10^, ^22^, ^23^ with 103 NMOSD patients (64 patients [62.1%] were AQP4 IgG seropositive patients) reported pre and posttreatment visual outcome.

**Figure 1:**
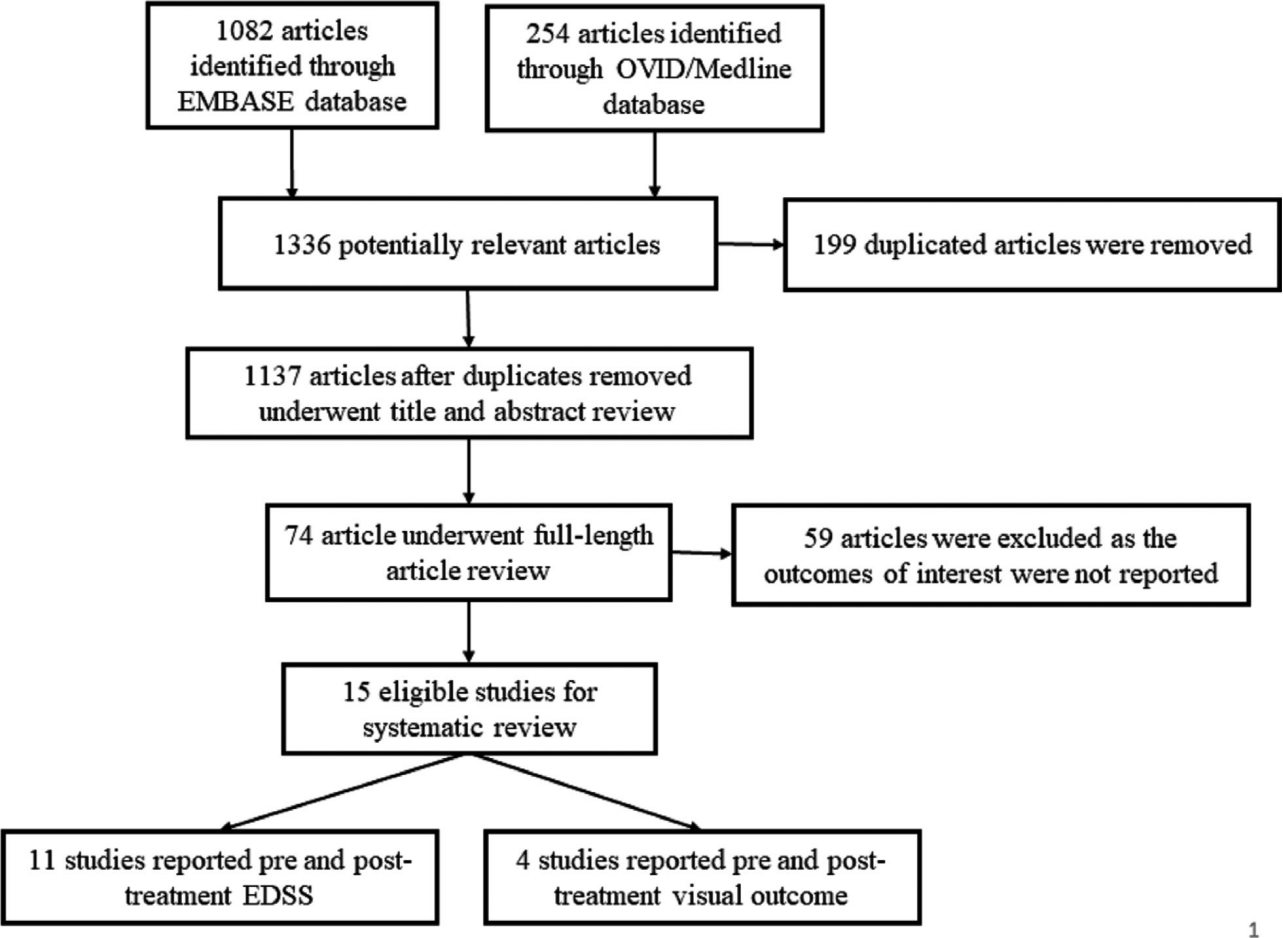
Flow‐chart of the literature review process for the evaluation of EDSS score.

The main characteristics and detailed information of the EDSS and VA studies are summarized in Table S1 and Table 1, respectively. The literature retrieval, review, and selection process are shown in Figure 1.

**Table 1:** Main characteristics of included studies reporting visual outcome.

Study	Year	Country	Study design	Recruitment of patients	Characteristics of NMOSD attacks	Criteria diagnosis of NMO/NMOSD/ON	Age of onset/diagnosis (mean (SD) [range]) (years)	Total number of NMOSD patients (number of AQP4‐IgG seropositive patients)	Number of patients with MOG‐IgG seropositivity	Number of patients received TPE (number of AQP4‐IgG seropositive patients)	No. of attack (No. of affected eye) in TPE receivers	TPE regimen in focused group	Other initial attack/relapse therapy in focused group	Assessment of treatment response and duration of follow‐up/treatment
Merle et al.^9^	2012	France	Ambispective, nonrandomized study	Database of University Hospital Center of Fort de France in Martinique from 1 January 1995, to 31 December 2010	Nonspecified acute attack of NMOSD	Wingerchuk 1999 NMO Criteria and Wingerchuk 2006 NMO Criteria	40.5 (14.8) [13–61]	52 (19)	N/A	16 (6)	N/A	In 2006, TPE (when available) was added to the corticosteroids Five daily consecutive TPEs	SPT (MP 2 g/day for 3–5 days)	6 months
Deschamps et al.^22^	2016	France	Retrospective cohort	Database of Hôpital Saint‐Louis, APHP Paris France from September 2010 to May 2015	All clinical attacks of ON	Wingerchuk 2006 NMO Criteria	37.4 (N/A) [17–61]	11 (8)	1 (of 3 in APQ4‐IgG seronegative)	11 (8)	15 (N/A)	TPE as a rescue therapy Time to TPE, median (mean; range), months 29 (32.3; 14–78) Number of TPE, median (mean; range), months 7 (6.4; 5–10)	All patients received SPT 1 g/day for at least 5–12 days Median interval between last SPT and first TPE was 3 days (mean 4.7; range 1–17)	Median (mean; range) 21 (24.5; 5–52) months
Mori et al.^23^	2018	Japan	Retrospective cohort	Database of Kobe University Hospital from March 2010 to September 2017	All clinical attacks of ON Number of ON episodes, mean (range) 1.2 (0–4)	IPND 2015 NMOSD Criteria	53 (N/A) [11–81]	9 (9)	N/A	9 (9)	N/A (15)	7 exchanges every other day for approximately 3 weeks Number of TPEs, mean 5.1 (range, 3–7)	SPT (MP 1 g/day for 3 days) before apheresis: TPE only (10 eyes), TPE plus IVIG (3 eyes), TPE plus IA (2 eyes)	1, 2, 3, and 4 weeks
Song et al.^10^	2019	China	Ambispective, nonrandomized study	Database of Southwest Eye Hospital from September 2015 to May 2018	3 patients were first attacked ON and the others were recurrent ON	N/A	36.13 (15.83) [12–67]	31 (28)	N/A	15 (N/A)	N/A (N/A)	Interval between SPT and TPE was 1.6 days TPE was performed 2–3 times a week. Number of TPE was 4 ± 1.37	3–5 days SPT	6 months

AQP4, aquaporin‐4; IA, immunoadsorption; IgG, immunoglobulin; IPND, International Panel for NMO Diagnosis; MOG, myelin oligodendrocyte glycoprotein; MP, methylprednisolone; NMO, neuromyelitis optica; NMOSD, neuromyelitis optica spectrum disorder; ON, optic neuritis; SPT, Steroid pulse therapy; TPE, therapeutic plasma exchange; VA, visual acuity.

### Improvement of EDSS

**Figure 2:**
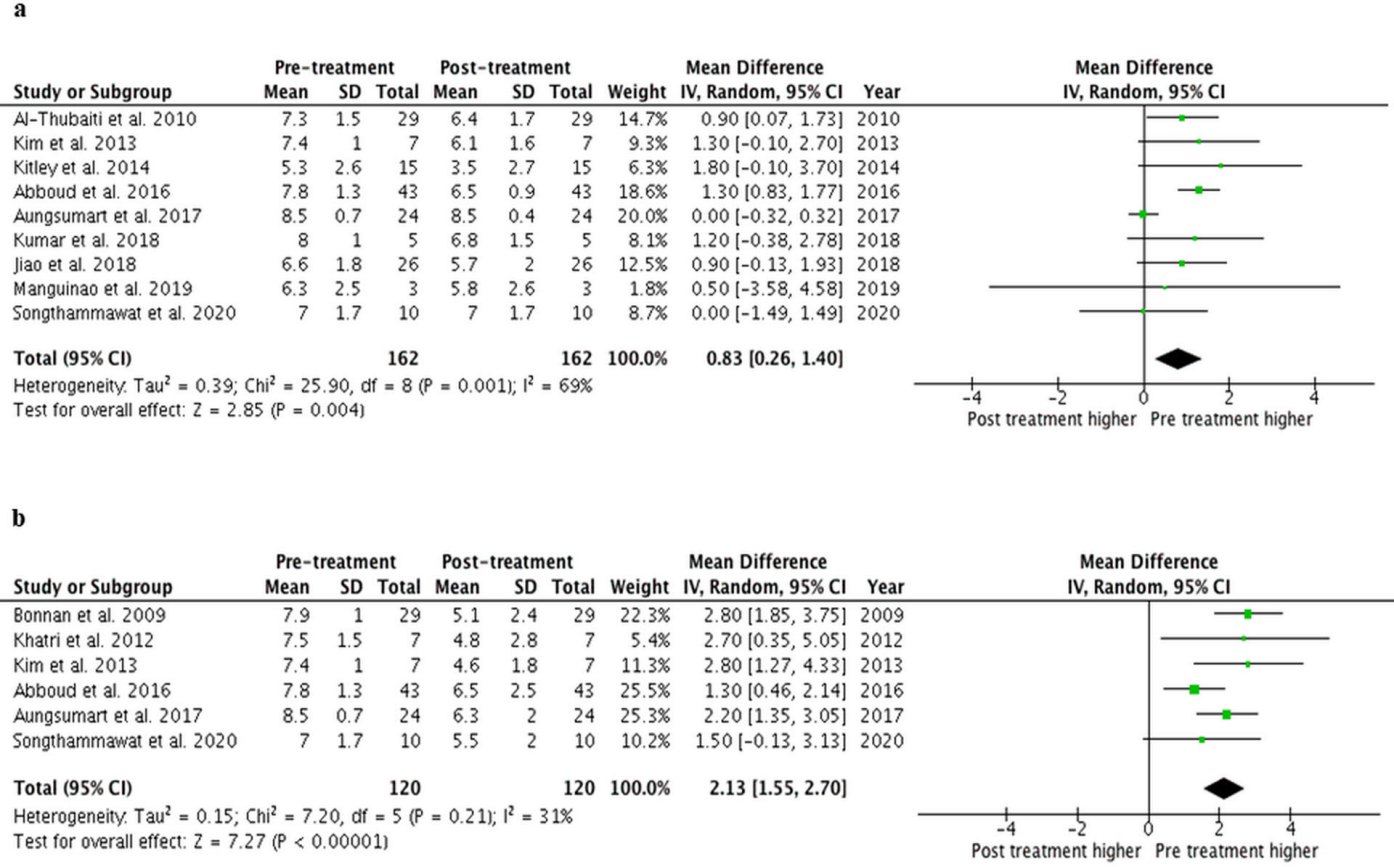
Forest plot of EDSS in patients with NMOSD after receiving TPE. (A) After TPE immediately. (B) At the follow‐up period. EDSS, Expanded Disability Status Scale; NMOSD, neuromyelitis optica spectrum disorder; TPE, therapeutic plasma exchange.

The included articles consisted of nine retrospective cohort studies,^5^, ^6^, ^14^, ^15^, ^16^, ^18^, ^19^, ^20^, ^21^ one prospective cohort study,^17^ and one randomized‐controlled study.^7^ All of the included studies reported EDSS pre and immediately posttreatment. In this study, the pretreatment EDSS was the EDSS scale measured on the first day of admission, whereas immediately posttreatment EDSS was the EDSS scale measured on the last day of TPE sessions for the two studies^19^, ^20^ or the one that measured on the last day of admission in the other two studies.^5^, ^7^ The meta‐analysis found that treatment with TPE during an acute attack is associated with a significantly decreased in EDSS with the pooled MD of 0.83 (95% CI, 0.26–1.40) comparing pretreatment to immediate posttreatment (Fig. 2A). The between‐study heterogeneity was moderate heterogeneity with I^2^ of 69%.

A total of six studies^5^, ^6^, ^7^, ^15^, ^16^, ^18^ also reported EDSS after long‐term follow‐up. The meta‐analysis found that treatment with TPE during an acute attack is also associated with a significantly decreased EDSS at 6 months to 1‐year follow‐up with the pooled MD of 2.13 (95% CI, 1.55–2.70) comparing pretreatment to posttreatment (Fig. 2B). The between‐study heterogeneity was low heterogeneity with I^2^ of 31%.

### Improvement of VA

A total of four studies^9^, ^10^, ^22^, ^23^ reported visual pre and posttreatment visual outcomes among patients with NMOSD who underwent TPE. Unfortunately, only one^10^ out of the four studies reported SD in association with mean LogMAR, and, therefore, the meta‐analysis could not be conducted. Nonetheless, all four studies consistently demonstrated the benefit of TPE with improved VA and/or LogMAR after treatment. Details on VA and/or LogMAR before and after treatment of the four studies are described in Table 2.

**Table 2:** Visual outcomes after treatment with TPE therapy.

Study	Visual outcomes	Mean/median pretreatment visual outcomes	Mean/median posttreatment visual outcomes
Merle et al.^9^	Visual acuity	20/400	20/50
Deschamps et al.^22^	LogMAR VA median (mean; range)	2 (1.84; 1 to 3)	0 (0.61; 0–3)
Mori et al.^23^	LogMAR, mean (range)	1.25 (0.30 to 2.30)	1 week; 1.00 (0.10–2.3) 2 weeks; 1.00 (0.10–2.3) 3 weeks; 0.97 (−0.08 to 2.3) 4 weeks; 0.82 (−0.08 to 2.3)
Song et al.^10^	LogMAR (mean ± SD)	1.94 ± 0.83	1.26 ± 0.66

LogMAR, Logarithm of the Minimum Angle of Resolution; N/A, not available; SD, standard deviation; TPE, therapeutic plasma exchange; VA, visual acuity.

### Adverse events

Of three studies^7^, ^19^, ^23^ that reported adverse effects related to TPE, 28 of 48 (58.3%) patients experienced a total of 36 adverse events. Specifically, there were seven events of allergy (19.4%), six events of hypofibrinogen (16.7%), five events of hypocalcemia (13.9%), four events of thrombosis‐related finding (11.1%), three events of low IgG level (8.3%), three events of transient hypotension (8.3%), two events of catheter‐related sepsis (5.6%), one event of thrombocytopenia (2.8%), one event of clinical bleeding (2.8%), one event of gastrointestinal reaction (2.8%), one event of nausea (2.8%), one event of arteriovenous fistula from double‐lumen insertion (2.8%), and one event of transmembrane pressure alarm with delay in a procedure (2.8%). Moreover, in the study by Manguinao et al.,^21^ two of nine NMOSD patients had unspecified complications due to TPE. All adverse events are shown in Table 3

**Table 3:** Adverse events in patients with acute NMOSD attack receiving TPE.

	Study			Total
	Jiao et al.^19^	Songthammawat et al.^7^	Mori et al.^23^	
Number of NMOSD patients experienced adverse effects (number of NMOSD patients received TPE)	11 (29)	8 (10)	9 (9)	28 (48)
Number of total adverse events	11	14	11	36 (%)
Noncatheter related				
Allergy	3	0	4	7 (19.4%)
Hypofibrinogen	0	6	0	6 (16.7%)
Hypocalcaemia	0	5	0	5 (13.9%)
Thrombosis‐related finding	0	1	3	4 (11.1%)
Low immunoglobulin level	1	0	2	3 (8.3%)
Transient hypotension	2	1	0	3 (8.3%)
Thrombocytopenia	1	0	0	1 (2.8%)
Clinical bleeding	1	0	0	1 (2.8%)
Gastrointestinal reaction	1	0	0	1 (2.8%)
Nausea	1	0	0	1 (2.8%)
Catheter related				
Catheter‐related sepsis	1	0	1	2 (5.6%)
Arteriovenous fistula from double‐lumen insertion	0	1	0	1 (2.8%)
Transmembrane pressure alarm with delay in a procedure	0	0	1	1 (2.8%)

NMOSD, neuromyelitis optica spectrum disorders; TPE, therapeutic plasma exchange.

### Evaluation for publication bias

**Figure 3:**
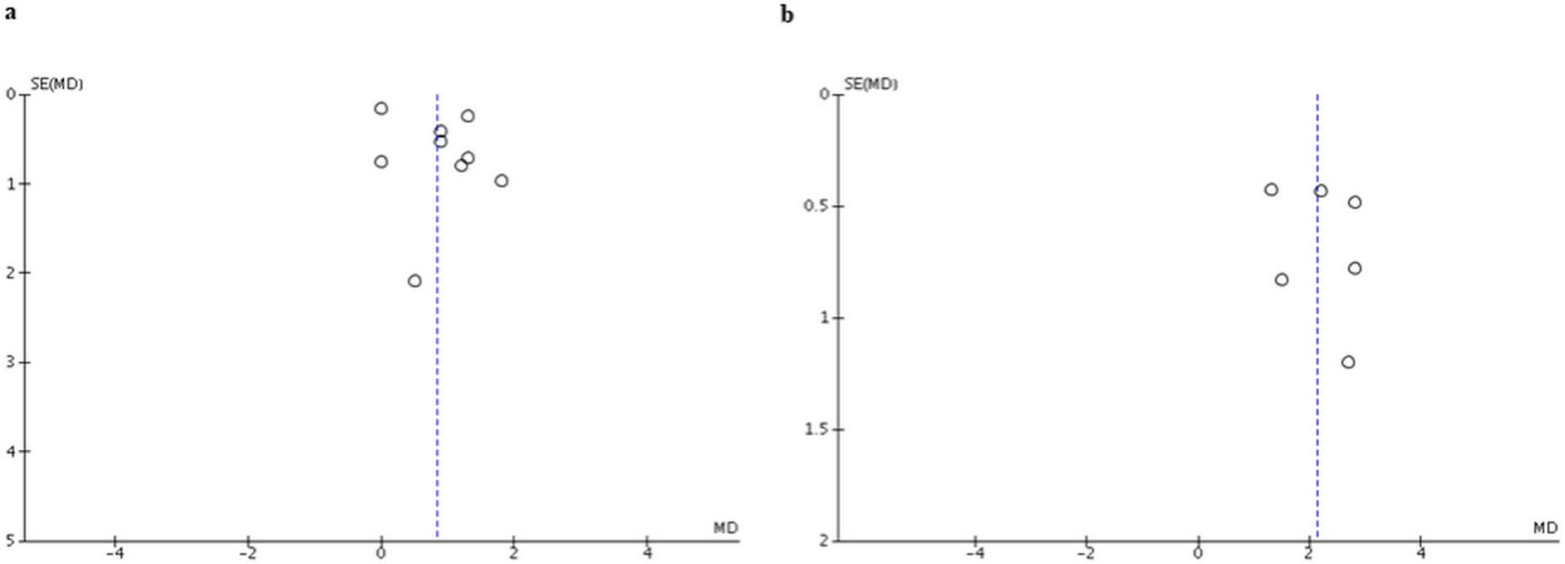
Funnel plot showing the incidence of EDSS of patients with NMOSD after receiving TPE. (A) After TPE immediately. (B) At the follow‐up period. EDSS, Expanded Disability Status Scale; NMOSD, neuromyelitis optica spectrum disorder; TPE, therapeutic plasma exchange.

A funnel plot was created to evaluate publication bias of EDSS after TPE immediately and at the follow‐up period. The plots were relatively symmetrical which was not suggestive of publication bias (Fig. 3).

## Discussion

This systematic review and meta‐analysis analyzed the data from all the available studies and found that TPE might be considered as an effective treatment for the NMOSD attack that is associated with the improvement of both disability status and visual outcomes. The disability benefit extended beyond just immediately after treatment to 6–12 months.

In 1999, Weinshenker et al. published a randomized, sham‐controlled, double‐masked study of TPE in patients with acute CNS Inflammatory demyelinating disease.^2^ This study included two NMOSD patients who failed to recover after treatment with high‐dose corticosteroids but subsequently showed dramatic improvement after TPE and remained stable without concomitant immunosuppressant. Therefore, the neurological improvement was explicitly contributed to the efficacy of TPE.

This study^2^ also recorded adverse events. There are mainly related to treatment and also included those related to the underlying demyelinating disease. Severely detrimental adverse events, however, were associated with the natural course of the underlying disease or incidental findings.

Our study revealed that greater than 60% of included patients were AQP4 seropositive patients and the majority of them showed good response to TPE. Although NMOSD patients with AQP4 seropositive status seem to have poorer visual outcome, poorer histological type of NMOSD lesion, higher relapse rate, and higher risk of treatment failure due to the reoccurrence of CD19^+^ B cells.^24^

To explain the improvement post‐TPE immediately, the elimination of various antibodies, complements, and cytokines which are the circulating pathogenic inflammatory mediators of NMOSD is probably the primary mechanism of TPE that leads to the observed beneficial effect.^4^, ^25^

The long‐term benefit after TPE may partly be explained by B‐cell depletion. Since AQP4 antibodies are produced by B cell differentiation to plasma cells and B cells are also potent antigen‐presenting cells for AQP4‐reactive pathogenic T cells,^26^ this current knowledge explains the scientific rationale for using B cell depletion therapy for long‐term prevention.

The depletion of B‐cell by TPE helps to prevent auto‐reactive B‐cell re‐expansion, which consequently helps to decrease AQP4 production in the long term like other B‐cell depletion therapy^26^, ^27^ Many studies have indicated that patient who underwent TPE had a lower level of AQP4 autoantibody titer.^16^, ^28^ However, the level of AQP4 autoantibody can rise during follow‐up due to the nature of the disease. Of all current knowledge, it may explain the reduction of disability in NMOSD patients after receiving TPE. Therefore, TPE, which is typically used in the prevention of disease, seems to be advantageous as a therapy for acute relapse as well.

There were some limitations in this systematic review and meta‐analysis that should be acknowledged. The most important limitation is the descriptive nature of the included studies that we could not confident whether the observed benefit is a result of TPE or co‐interventions. Baseline characteristics of participants and TPE protocol also varied considerably across the studies. Therefore, how exactly to perform TPE and whether to use it as rescue therapy in steroid nonresponder or as concomitant therapy with intravenous corticosteroid or intravenous IgG is still a matter of debate. Moreover, the role of TPE whether to use in the first attack or to use in relapse treatment is still inconclusive since the included studies are limited. Last, it is still possible that publication bias in favor of positive studies may have been present even though the relatively symmetrical funnel plots as the number of the included studies is relatively small, which jeopardizes the interpretability of the plots.

## Conclusions

This systematic review and meta‐analysis found that the utilization of TPE during the NMOSD attack is associated with a significantly improved disability status immediately after treatment and during follow‐up. However, the descriptive nature of the included studies was the main limitation of this meta‐analysis.

## Author Contributions

P.K., P.U. and J.J. contributed to drafting and revising the manuscript for content, including medical writing for content, study concept and design, analysis and interpretation of data, and acquisition of data. S.S. contributed to aiding in drafting and revising the manuscript for content, including medical writing for content, study concept and design, analysis and interpretation of data, and acquisition of data. S.S. and N.P. contributed to drafting and revising the manuscript for content, analysis, and interpretation of data. J.J. also contributed to study supervision.

## Conflict of Interests

Ms. Kosiyakul P declares that there is no conflict of interest. Mr. Songwisit S declares that there is no conflict of interest. Dr. Ungprasert P declares that there is no conflict of interest. Dr. Siritho reports grants from Merck Serono, Pacific Healthcare (Thailand), Menarini (Thailand), Biogen Idec, UCB (Thailand), Eisai Inc, Sanofi‐Aventisa, Terumo BCT, and Novartis for travel and speaker honoraria, outside the submitted work. Dr. Prayoonwiwat reports grants from Bayer Schering Pharma, Eisai Inc, UCB, Thailand, Merck Serono, Pfizer Pharmaceutical Company Limited, Novartis, and Sanofi‐Aventis for travel and received speaker honoraria, outside the submitted work. Dr. Jitprapaikulsan J. declares that there is no conflict of interest.

## Disclaimers

The authors state that the views expressed in the submitted article are not his or her own and not an official position of the institution or funder.

Comments: please edit Data S1. Search term from EMBASE and OVID

## Supporting information


**File S1.** PRISMA 2009 flow diagram.

**Table S1.** Major characteristics of included studies reporting changes of EDSS outcome in the meta‐analysis.

**Data S1.** Search term from EMASE and OVID.
