# Supplementary material for: Effect of plasma exchange in neuromyelitis optica spectrum disorder: A systematic review and meta‐analysis
Source: Ann Clin Transl Neurol. 2020 Sep 21;7(11):2094–102. doi: 10.1002/acn3.51203 (PMC7664276; doi:10.1002/acn3.51203)
Supplement: Supplementary file 1 — File S1. PRISMA 2009 flow diagram. [file ACN3-7-2094-s001.doc]

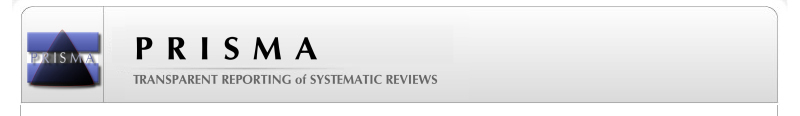
**PRISMA 2009 Flow Diagram**

**Screening**

**Included**

**Eligibility**

**Identification**

Records identified through database searching
(n = 1336)

Additional records identified through other sources
(n = 0)

Records after duplicates removed
(n = 1137)

Records screened
(n =1137)

Records excluded
(n =1063)

Full-text articles assessed for eligibility
(n =74)

Full-text articles excluded, with reasons
(n =59)

Studies included in qualitative synthesis
(n = 15)

Studies included in quantitative synthesis (meta-analysis)
(n = 11)
