# Supplementary material for: Effect of plasma exchange in neuromyelitis optica spectrum disorder: A systematic review and meta‐analysis
Source: Ann Clin Transl Neurol. 2020 Sep 21;7(11):2094–102. doi: 10.1002/acn3.51203 (PMC7664276; doi:10.1002/acn3.51203)
Supplement: Supplementary file 2 — Table S1. Major characteristics of included studies reporting changes of EDSS outcome in the meta‐analysis. [file ACN3-7-2094-s002.docx]

**Supplementary Table** Major characteristics of included studies reporting changes of EDSS outcome in the meta-analysis

| **Study** | **Year** | **Country** | **Study design** | **Recruitment of patients** | **Characteristics of NMOSD attacks** | **Total number of TM attacks** | **Diagnostic criteria of NMO / NMOSD** | **Age of onset/diagnosis**  **(mean (SD) [range] ) (years)** | **Total number of NMOSD patients (number of patients with AQP4-IgG seropositivity)** | **Total number of patients with MOG-IgG seropositivity** | **TPE regimen** | **Other initial attack/relapse therapy** | **Assessment of treatment response and duration of follow-up/treatment** |
| --- | --- | --- | --- | --- | --- | --- | --- | --- | --- | --- | --- | --- | --- |
| Bonnan et al.^6^ | 2009 | France | Retrospective cohort | Database of Hôpital Zobda Quitman d from 1982 to 2008 | - Single or relapsing TM  - Patients who received PE  during ≥1 spinal attack : Patients who never received PE = 25:18 | 96 (number of TM received TPE = 29) | Wingerchuk 1999 NMO Criteria | 34 (14)  [14–82] | 43 (34) | N/A | - TPE was started as soon as possible 2 days after attack. TPE was never initiated as a delayed rescue treatment after a standard steroid treatment failure.  - TPE was carried out daily for 5 days | 3–10 g of IVMP | > 6 months |
| Al- Thubaiti et al.^14^ | 2010 | Canada | Retrospective cohort | Database of University of British Colombia from 2001 to 2009 | N/A | N/A | N/A | 44.01 (9.44)  [22-60] | 29 (N/A) | N/A | N/A | High dose steroid pulse | N/A |
| Khatri et al.^15^ | 2012 | USA | Retrospective cohort | N/A | All clinical attacks of TM with maintenance TPE | Total number of NMOSD patients presented with TM =7 (number of patients received TPE = 7) | Wingerchuk 2006 NMO Criteria | 31 (N/A)  [17–60] | 7 (7) | N/A | - Patients undergone TPE when no marked or moderate improvement in response to 7 days of high‐dose corticosteroids after an exacerbation TPE resulted in approximately 3 L of plasma removal for a 60‐kg person.  - At induction, the patients underwent TPE three times per week for 2 weeks, then two times a week for 2 weeks, then once a week for 3–5 weeks.  - After this induction, the patients experienced a TPE taper of gradually declining TPE frequency as dictated by the patient's clinical condition. This empirical TPE taper was continued as long as patients remained stable, ultimately resulting in patients undergoing TPE once every 3–12 weeks | High‐dose corticosteroids | > 6 months |
| Kim et al.^16^ | 2013 | Korea | Retrospective cohort | Patients with NMO/ NMOSD were identified from March 2010 to September 2011 | Acute attack of NMOSD, prior to treating an attack with TPE | Total number of NMOSD patients presented with TM = 5 (number of patients received TPE = 5) | Wingerchuk 2006 NMO Criteria or Revised 2007 NMO Criteria | Median age at the time of TPE was 40 [range 12-53] | 15 (N/A) | N/A | - TPE was performed when severe disability was sustained or worsened after high-dose IVMP therapy as indicated by EDSS scores ≥7.0 or a visual acuity worse than 20/200. | High-dose IVMP therapy (1 g for 5 days) | 6 months |
| Kitley et al.^17^ | 2014 | England | Prospective cohort | Database of Oxford clinical NMO service from January 1, 2010 to April 1, 2013 | First attack of NMOSD | 21 (number of TM received TPE = 21) | N/A | 44.86  (14.8) [N/A] | 29 (20) | 9 | - High proportion of patients in each group received corticosteroids in the acute setting (100% MOG-Ab positive vs 75% AQP4-Ab positive; P = 0.15) and similar proportions additionally underwent plasma exchange (TPE) (33% vs 25%; P = 0.68)  -Plasma exchange was then instituted if patients showed no or limited clinical improvement within 7 days after corticosteroid | IVMP 1 g/d for 3 days, extending to 5 days if no clinical improvement occurred, was used (with the exception of 1 patient in each group treated with oral methylprednisolone, 500 mg, daily for 5 days) | N/A |
| Abboud et al.^5^ | 2016 | USA | Retrospective cohort | Database of Johns Hopkins Hospital from 2005 to 2013 | Relapses of ON or TM | 33 (number of TM received TPE = N/A) | Wingerchuk 2006 NMO Criteria | 41.6 (18.2) | 43 (27) | N/A | - In the IVMP + TPE group, all patients received 5 to 7 sessions of TPE in the second week of presentation following a five-day trial of IVMP.  - One to 1.5 volumes of plasma were exchanged at each session. | High-dose IVMP 1g/day for five days starting on day 1 of admission | 1 year |
| Aungsumart et al.^18^ | 2017 | Thailand | Retrospective cohort | N/A | Nonspecified acute attack of NMOSD | 16 (number of TM received TPE = 16) | IPND 2015 NMOSD Criteria | Median 41  (IQR 34–48) | 24 (20) | N/A | - Plasma exchange was performed when severe disability was sustained or worsened after IVMP (1 g for 5 days) as indicated by EDSS scores ≥7.0 in patients who presented with myelitis or a visual acuity worse than 20/200 in patients who presented with optic neuritis.  - Plasma exchange was performed every other day for 5 treatments. | 1 g of IVMP for 5 days. | 6 months |
| Jiao et al.^19^ | 2018 | China | Retrospective cohort | Database of China-Japan Friendship Hospital from October 2010 to July 2017 | Relapses of ON or TM | 16 (number of TM received TPE = 16) | Wingerchuk 2006 NMO Criteria and IPND 2015 NMOSD Criteria | 39.44 (18.54) [15-78] | 29 (23) | N/A | - Patients received TPE if a limited response or deterioration in neurologic conditions was observed after an initial standard dose of IVCS or IVIG treatment, or if EDSS score ≥6 was determined by at least 2 physicians on admission.  - All candidates received 2 to 7 sessions of TPE every other day  - One volume of plasma (~2-L plasma volume) was exchanged at each session. | 23 patients received IV corticosteroids prior to TPE, 6 of them also received IVIG | 1 month |
| Kumar et al.^20^ | 2018 | India | Retrospective cohort | Database of Dayanand Medical College and Hospital, Ludhiana, Punjab, India from January 2013 to December 2016 | Nonspecified acute attack of NMOSD | Total number of NMOSD patients presented with TM = 5 (number of patients received TPE = 5) | Wingerchuk 2006 NMO Criteria | 52.5 (N/A)  [36-69] | 5 (5) | N/A | - TPE for acute exacerbations after being refractory to high-dose steroid treatment which was given for 5 days and also repeated if there was worsening of symptoms for the next 5 days.  - TPE was scheduled preferably on alternate-day intervals for 8 to 10 days estimated plasma volume (EPV).  - A simple means of estimating, the EPV can be calculated EPV = (0.65 × wt [kg]) × [1 − Hcv] mean time of start of TPE in the acute attack was 18.6 days, the mean number of TPE session was 4.4 (standard deviation [SD] ±1.2), the mean volume of plasma exchange was 2875 ml (SD ± 125) and mean time duration of a session was 270 min (SD ± 35). | - High-dose steroid treatment  -Some of these patients also received other disease-modifying immune-modulatory therapies such as cyclophosphamide and rituximab during their course of the disease. | N/A |
| Manguinao et al.^21^ | 2019 | USA | Retrospective cohort | Database of Pediatric MS Center at University of California, San Francisco (UCSF) from November 2006 to April 2018. | Nonspecified acute attacks of NMOSD | N/A | N/A | Median 10.5 years  [range 2–17] | 7 (N/A) | N/A | - TPE as a second- or third-line therapy.  - Number of plasma exchange sessions, n = 25, median 5 (range 3–10)  - Plasma volume ratio exchanged per cycle, n = 15 median 1.0 (range 1.0–1.5) | - IV high-dose pulse steroids prior to TPE with median treatment duration before TPE of 5 days (range 1–10)  (n = 24). | N/A |
| Songthammawat et al.^7^ | 2020 | Thailand | Randomized, controlled study | Database of Siriraj Hospital, Bangkok, Thailand from January 2016 to December 2018 | Nonspecified acute attacks of NMOSD | N/A | IPND 2015 NMOSD Criteria | 37.9 (14.7)  [N/A] | 10 (10) | N/A | - TPE was given only if a patient was not responding to the IVMP treatment at day 7 after the first day of IVMP initiation. 5 patients were treated with IVMP + TPE regimen; a combination of simultaneous IVMP 1 g/D for 7 consecutive days and centrifugal TPE method.  - TPE was performed on alternate days for 5 sessions, starting on the first day of IVMP treatment, with 1 liter of total plasma volume exchanged for a similar volume of 5% albumin. | 5 patients were treated with IVMP add-on TPE regimen; IVMP 1 g/d for 7 consecutive days and then subsequently TPE | 6 months |

Abbreviations: AQP4, aquaporin-4; EDSS, Expanded Disability Status Scale; IgG, immunoglobulin; IPND, International Panel for NMO Diagnosis; IVCS, intravenous corticosteroid (methylprednisolone or dexamethasone); IVIG, intravenous immunoglobulin; IVMP, intravenous methylprednisolone; MOG, myelin oligodendrocyte glycoprotein; N/A, not available; NMO; neuromyelitis optica; NMOSD, neuromyelitis optica spectrum disorder; ON, optic neuritis; TPE, therapeutic plasma exchange; TM, transverse myelitis
