# Supplementary material for: Effect of plasma exchange in neuromyelitis optica spectrum disorder: A systematic review and meta‐analysis
Source: Ann Clin Transl Neurol. 2020 Sep 21;7(11):2094–102. doi: 10.1002/acn3.51203 (PMC7664276; doi:10.1002/acn3.51203)
Supplement: Supplementary file 3 — Data S1. Search term from EMASE and OVID. [file ACN3-7-2094-s003.doc]

**Supplementary data**

**Search terms**

General search and Medical Subject Headings (MeSH) terms were used as follows:

**EMBASE**

1. ‘neuromyelitis optica’/exp OR ‘neuromyelitis optica’

2. ‘myelooptic neuropathy’/exp OR ‘myelooptic neuropathy’

3. ‘devic disease’/exp OR ‘devic disease’

4. ‘aqp 4’

5. ‘aqp4’

6. ‘aquaporin 4’/exp OR ‘aquaporin 4’

7. ‘aquaporin4’

8. ‘aquaporin 4 antibody’/exp OR ‘aquaporin 4 antibody’

9. ‘aquaporin 4 autoantibody’/exp OR ‘aquaporin 4 autoantibody’

10. #1 OR #2 OR #3 OR #4 OR #5 OR #6 OR #7 OR #8 OR #9

11. ‘plasma exchange’/exp OR ‘plasma exchange’

12. ‘plasmapheresis’/exp OR ‘plasmapheresis’

13. ‘apheresis’/exp OR ‘apheresis’

14. plex

15. #11 OR #12 OR #13 OR #14

16. #10 AND #15

**OVID**

1. Neuromyelitis Optica.mp. or Neuromyelitis Optica/

2. NMO.mp.

3. myelooptic neuropathy.mp.

4. optic neuritis.mp. or Optic Neuritis/

5. devic.mp.

6. devic disease.mp. or Neuromyelitis Optica/

7. demyelinating autoimmune disease.mp.

8. autoimmune encephalitis.mp.

9. aquaporinopathy.mp.

10. Aquaporin 4/ or aquaporin 4.mp.

11. AQP4.mp.

12. demyelinating.mp.

13. nmosd.mp.

14. 1 or 2 or 3 or 4 or 5 or 6 or 7 or 8 or 9 or 10 or 11 or 12 or 13

15. plasma exchange.mp. or Plasma Exchange/

16. plasmapheresis.mp. or Plasmapheresis/

17. 15 or 16

18. 14 and 17
